# A Quick Method to Assess Airway Distensibility in Mice

**DOI:** 10.1007/s10439-024-03518-9

**Published:** 2024-04-15

**Authors:** Rebecka Gill, Magali Boucher, Cyndi Henry, Ynuk Bossé

**Affiliations:** Institut Universitaire de Cardiologie et de Pneumologie de Québec (IUCPQ)–Université Laval, 2725, Chemin Sainte-Foy, Quebec, QC G1V 4G5 Canada

**Keywords:** Respiratory mechanics, Lung physiology, Impedance, Computational model, Airway resistance

## Abstract

Airway distensibility is defined as the ease whereby airways are dilating in response to inflating lung pressure. If measured swiftly and accurately, airway distensibility would be a useful readout to parse the various elements contributing to airway wall stiffening, such as smooth muscle contraction, surface tension, and airway remodeling. The goal of the present study was to develop a method for measuring airway distensibility in mice. Lungs of BALB/c and C57BL/6 mice from either sex were subjected to stepwise changes in pressure. At each pressure step, an oscillometric perturbation was used to measure the impedance spectrum, on which the constant-phase model was fitted to deduce a surrogate for airway caliber called Newtonian conductance (*G*_*N*_). The change in *G*_*N*_ over the change in pressure was subsequently used as an index of airway distensibility. An additional group of mice was infused with methacholine to confirm that smooth muscle contraction changes airway distensibility. *G*_*N*_ increased with increasing steps in pressure, suggesting that the extent to which this occurs can be used as an index of airway distensibility. Airway distensibility was greater in BALB/c than C57BL/6 mice, and its variation by sex was mouse strain dependent, being greater in female than male in BALB/c mice with an inverse trend in C57BL/6 mice. Airway distensibility was also decreased by methacholine. This novel method swiftly measures airway distensibility in mice. Airway distensibility was also shown to vary with sex and mouse strain and to be sensitive to the contraction of smooth muscle.

**Supplementary Information:**

The online version contains supplementary material available at 10.1007/s10439-024-03518-9.

## Introduction

A slew of elements influences the mechanics of the airway wall. These include the level of airway smooth muscle activation, the magnitude of the surface tension, and the extent and nature of structural changes (i.e., remodeling) that sometimes arise in diseased airway wall. Many of these elements are also altered, or likely to be altered, in animal models of respiratory diseases. Yet the mechanics of the airway wall for the ensemble of airways within the lung are not easy to measure in vivo. A quick and accurate method to appraise airway wall mechanics would be extremely convenient, especially if it is applicable to any animal model of respiratory diseases and repeatable within the same animal.

Recently, Robichaud et al. have developed a method for measuring airway compliance in mice, abbreviated *C*_aw_ [1]. The method is more precisely embedded in a longer procedure that was initially designed to assess lung volumes in mice, including residual volume and total lung capacity. It involves degassing the lung by apnea after a 5-min period of ventilation with 100% oxygen. The collapsed lung is then re-inflated at a constant flow. The pressure first rises rapidly during re-inflation with only a small change in volume until it reaches the opening pressure, where volume abruptly increases due to airway recruitment. The measurement of *C*_aw_ is based on the rationale that the small change in volume prior to the opening pressure is dictated by the compliance of the wall in all but the small closed airways within the lung. Although useful and very convenient in experiments where lung volumes are already measured, the method is technically challenging, takes about 13 min, and is lethal; the latter implying that it cannot be measured twice in the same mouse before and after an intervention.

In human subjects, different methods were developed to assess airway distensibility [2]. For example, airway caliber can be measured directly at different static lung pressures or volumes using computed tomography to subsequently calculate the rate at which it changes across a given range of pressure or volume [3–10]. A simpler method rather harnesses the many coveted features of oscillometry [11–16], including its ease and rapidity of implementation, its harmless nature (non-invasive and non-radiating), and many of its readouts capturing specific characteristics of not only one but all open airways within the lung. This oscillometric method was first developed by Brown et al. [12]. It essentially consists of measuring respiratory system conductance at a specific frequency (typically 5 Hz), a surrogate for airway caliber, at varying lung pressures or volumes. The rate of change in conductance over the change in lung pressure or volume then reflects airway distensibility, a readout sensitive to changes in the mechanical properties of the wall in the ensemble of open airways within the lung. An equivalent method to measure airway distensibility in mice is currently lacking. This would be important for translational purposes, as mice could then be used for testing experimental interventions, including drugs, which target elements contributing to airway wall stiffness, such as smooth muscle contraction, surface tension, and airway wall remodeling. The goal of the present study was to develop a rapid oscillometric method for measuring airway distensibility in mice in a way that allows repeated measurements within the same mouse.

## Materials and Methods

### Mice

Ten female and 10 male BALB/c mice (Charles River, Saint-Constant, Canada), together with 10 female and 16 male C57BL/6 mice (Jackson, Bar Harbor, MA, USA), were studied at 8 weeks of age. They were provided food and water ad libitum at all times. All procedures were approved by the Committee of Animal Care of *Université Laval* following the guidelines from the Canadian Council on Animal Care (protocol 2020-652).

### Mechanical Ventilation

Mice were anesthetized and put under general analgesia using ketamine (100 mg/kg) and xylazine (10 mg/kg), and then tracheotomized and connected to the flexiVent (FX Module 2, SCIREQ, Montreal, QC, Canada) as previously described [17, 18]. They were ventilated mechanically at a tidal volume of 10 mL/kg with an inspiratory-to-expiratory time ratio of 2:3 at a breathing frequency of 150 breaths/min and with a positive end-expiratory pressure of 3 cmH_2_O. Once the ventilation was underway, mice were paralyzed by injecting 100 and 300 µL of pancuronium bromide (0.1 mg/kg) intramuscularly and intraperitoneally, respectively, to avoid spontaneous breathing during the procedure.

### Respiratory Mechanics and Airway Distensibility

**Fig. 1:**
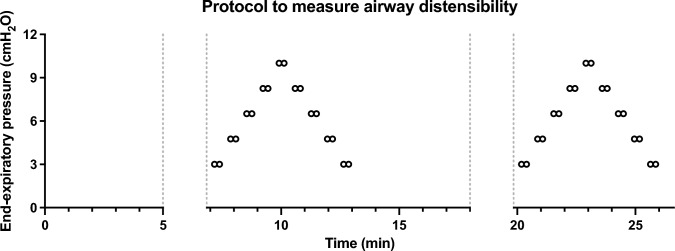
Protocol to measure airway distensibility in BALB/c mice. The lung of anesthetized and paralyzed mice cannulated via a tracheotomy was subjected to four sequential inflating steps in end-expiratory pressure followed by four sequential deflating steps. This protocol was repeated twice at about 7-min interval. Each circle represents a time point where Newtonian resistance (*R*_*N*_) was measured using the Quick Prime-3 (i.e., an oscillometric perturbation measuring the impedance spectrum of the respiratory system, on which the constant-phase model is then fitted to deduce *R*_*N*_). At each end-expiratory pressure, the Quick Prime-3 was actuated twice at an interval of 13 s. At any other time, mice were mechanically ventilated as described in “Methods.” The breaks on the *x*-axis between horizontal gray-dotted lines represent periods where baseline respiratory mechanics were measured, each time including two deep inflations to 35 cmH_2_O. The protocol was identical in C57BL/6 mice, except that the maximal pressure was set to 12 instead of 10 cmH_2_O

Respiratory mechanics and airway distensibility were also measured with the flexiVent. Baseline respiratory mechanics were first assessed by oscillometry before and after two deep inflations to 35 cmH_2_O. The lungs were then subjected to stepwise changes in inflating pressure, starting at 3 cmH_2_O. The protocol is illustrated in Fig. 1. The whole protocol was repeated twice at an interval of 6 min 20 s to measure reproducibility. For each protocol, there was four inflating steps, followed by four deflating steps, each step lasting 41 s. Preliminary results demonstrated that staying longer at each step was not affecting significantly the results. Each ascending or descending step of pressure was covering 1/4 of the pressure range. The pressure range was 7 cmH_2_O (3 to 10 cmH_2_O) for BALB/c mice and 9 cmH_2_O (3 to 12 cmH_2_O) for C57BL/6 mice. The pressure range was different between mouse strains because preliminary results demonstrated that the approximately linear change in Newtonian resistance (*R*_*N*_) caused by inflating pressure occurs over a smaller range in BALB/c compared to C57BL/6 mice. This is consistent with the stiffer lung of C57BL/6 compared to BALB/c mice [17].

At every pressure step, respiratory mechanics were evaluated by probing the lung with a small-amplitude oscillometric perturbation, colloquially called the Quick Prime-3. This was done twice at 10 s interval, the first one occurring 20 s after each step in pressure. The Quick Prime-3 is an input flow perturbation made of 13 sine waves of mutually prime frequencies, each with a different amplitude and phase, allowing the impedance spectrum of the respiratory system (i.e., the resistance and reactance at every tested frequency) to be calculated from the resulting output pressure [19]. The impedance spectrum was then analyzed using a computational model called the constant-phase model [20]:

$Z\left( \omega \right) = R_{N} + {{\left( {G \, - \, iH} \right)} \mathord{\left/ {\vphantom {{\left( {G \, - \, iH} \right)} {\omega^{\alpha } }}} \right. \kern-0pt} {\omega^{\alpha } }},$ where *Z* is impedance, *ω* is angular frequency, *i* is the imaginary unit, and *α* is 2/*π* arctangent of *H*/*G*. The model includes three parameters. One is called *G*, which reflects the tissue resistance of the lung and the chest wall [21–23], although it is also sensitive to small airway narrowing heterogeneity [24]. Another one is *H*, which reflects the elastance of the whole lung and is, thus, sensitive to both the accessible (i.e., reachable from the trachea) volume of the lung and the tissue stiffness of the lung and the chest wall [21, 22]. The other one is Newtonian resistance (*R*_*N*_), which reflects the resistance to airflow in conducting airways. *R*_*N*_ was then inverted (1/*R*_*N*_) to get Newtonian conductance (*G*_*N*_). The change in conductance with inflating pressure was previously shown to be a good proxy for the change in airway volume [12]. The rate of change of *G*_*N*_ with inflating pressure, thus, represents an index of airway distensibility, reflecting the ease whereby the caliber of the ensemble of open airways is dilating in response to inflating pressure.

### Methacholine Challenge

Six male C57BL/6 mice were used to assess the effect of methacholine on airway distensibility. Mice were prepared as described above, except that a 25-gauge cannula was inserted into the right jugular vein to deliver methacholine intravenously. The protocol to assess airway distensibility was performed twice, first without methacholine and then with infused methacholine. The methacholine was delivered with an infusing pump (Pump 11 Pico Plus Elite Dual, Harvard Apparatus, USA) at a rate of 21.7 µg/min/kg (mouse weight ranged between 24.7 and 28.2 g). It was delivered during 6 min 20 s before the assessment of airway distensibility.

### Data Analysis

Individual data are presented, together with means ± standard deviations (SD). Intra-class correlation coefficients (ICC) were calculated to measure reproducibility of airway distensibility between the first and the second protocols. Paired *t* tests were also used to compare values of airway distensibility between the first and the second protocols. In addition, Bland–Altman analyses were used to assess the level of agreement in values of airway distensibility between the first and the second protocols. Airway distensibility was then analyzed by two-way ANOVA to measure the effect of the mouse strain, sex, and their interaction. When the interaction was significant, it was followed by a Sidak’s multiple comparisons test to compare between sexes within each mouse strain. Finally, the effect of methacholine on airway distensibility was measured by a paired *t* test. All statistical analyses were performed with Prism (version 10.1.0, GraphPad, San Diego, CA). Differences with *p* < 0.05 were considered statistically significant.

## Results

Each mouse was successfully subjected to the protocol twice (Fig. 1) without technical difficulties. The value of *R*_*N*_ between the first and the second Quick Prime-3 at each pressure step was virtually identical (data not shown). The two values were, thus, averaged to obtain one *R*_*N*_ value per pressure and *R*_*N*_ was then inverted to obtain *G*_*N*_.

**Fig. 2:**
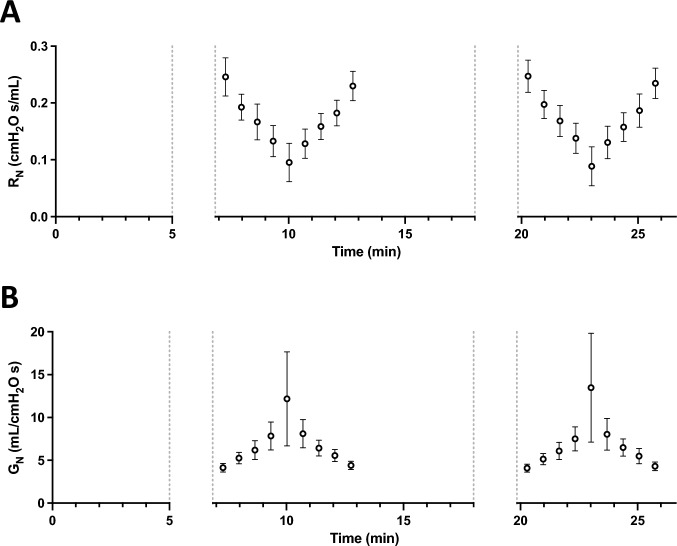
**A** The changes in Newtonian resistance (*R*_*N*_) over time during the step changes in end-expiratory pressure shown in Fig. 1. Results (means ± SD) are from BALB/c mice (*n* = 10 males and 10 females combined). The two values of *R*_*N*_ obtained at each pressure step were first averaged for each mouse, and values for all mice were then compiled to obtain a mean value per pressure step. **B** The corresponding changes in Newtonian conductance (*G*_*N*_) during the same experiments

**Fig. 3:**
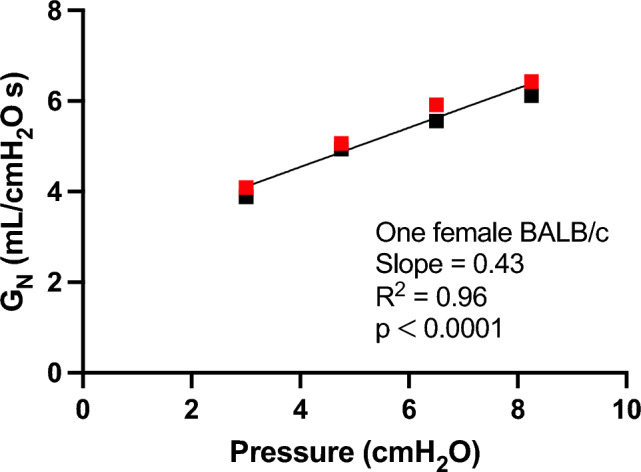
Calculating airway distensibility in one mouse. The Newtonian conductance (*G*_*N*_) was plotted against the end-expiratory pressure. The graph shows data from one female BALB/c mouse during the stepwise changes in pressure shown in Fig. 1. Only the first four ascending points (black) and the last four descending points (red) were used to calculate airway distensibility, omitting the data at the highest pressure point because it was not linearly related to the other (see Fig. 2B). A linear regression was then fitted to assess the relationship between *G*_*N*_ and pressure. The slope of this linear regression defines the extent to which *G*_*N*_ is increasing with inflating pressure. Since *G*_*N*_ reflects airway conductance to airflow and its changes are mainly determined by changes in airway caliber, this slope is also a surrogate for airway distensibility

As expected, *R*_*N*_ decreased with increasing pressure steps and then increased with decreasing pressure steps (Fig. 2A). Concordantly, *G*_*N*_ increased with increasing pressure steps and then decreased with decreasing pressure steps (Fig. 2B). This is consistent with the airway dilating effect of lung inflation and the airway narrowing effect of lung deflation. In contrast to *R*_*N*_ though, the changes in *G*_*N*_ were not linear over the entire range of pressure (Fig. 2). At the highest pressure point (10 and 12 cmH_2_O for BALB/c and C57BL/6 mice, respectively), there was a clear departure from linearity with a sudden disproportional increase in *G*_*N*_ that was also a lot more variable between mice. In order to obtain a unique value of airway distensibility per mouse across the widest range of lung pressure possible, only data points within the approximately linear range were used to calculate distensibility. More precisely, the first four points during the ascending portion and the last four points during the descending portion of the stepwise changes in pressure were used. For each mouse, these *G*_*N*_ values were plotted over pressure, and a linear regression was then traced to calculate the slope that best defines the relationship between *G*_*N*_ and pressure over this pressure range (Fig. 3). Since the changes in *G*_*N*_ is mainly determined by changes in airway caliber, this slope describes the ease whereby the caliber of airways is expanding within this range of pressure. It is thus an index of airway distensibility, with a greater slope indicating a greater distensibility.

**Fig. 4:**
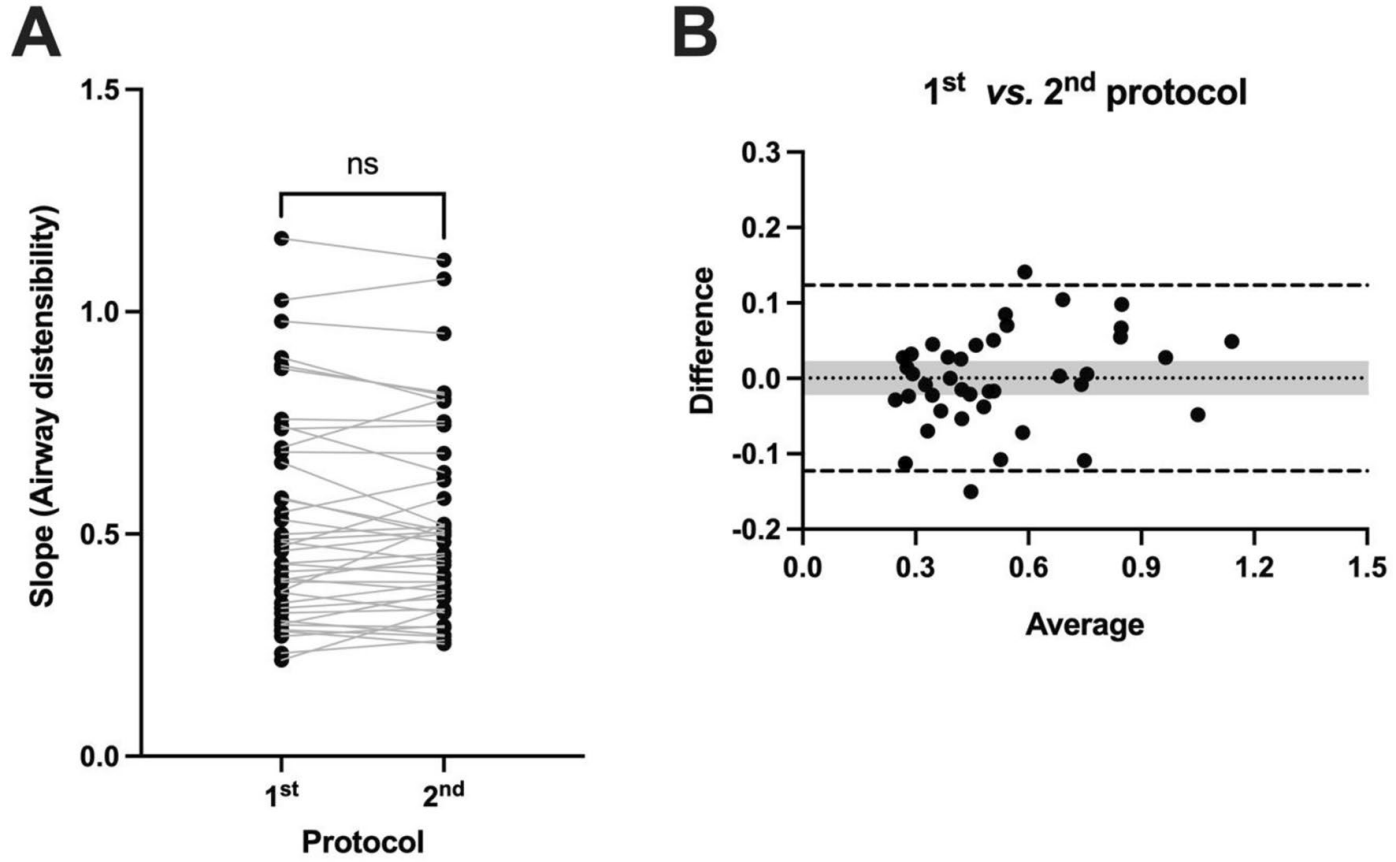
Reproducibility of airway distensibility. **A** Airway distensibility, measured as described in Fig. 3, was assessed twice in each mouse as described in the series of two protocols illustrated in Fig. 1. For each mouse, a line is connecting values obtained in the first and the second protocols. Based on a paired *t* test, airway distensibility between the first and the second protocols was not significantly different (ns). **B** Bland–Altman plot comparing the values of airway distensibility between the first and the second protocols. Each symbol represents one mouse, relating the difference between the two protocols (airway distensibility measured during the 1st protocol minus airway distensibility measured during the 2nd protocol) on the *y*-axis with the average of both protocols (the sum of airway distensibility in the 1st and the 2nd protocol divided by two) on the *x*-axis. The dotted line is the bias, the dashed lines are the 95% upper and lower limits of agreement, and the gray-shaded area is covering the upper and lower 95% confidence intervals for the bias. *n* = 40 (10 female BALB/c, 10 male BALB/c, 10 female C57BL/6 & 10 male C57BL/6)

This measure of distensibility was reproducible within mice. In fact, the ICC was 0.96, which represents an excellent reproducibility [25]. The distensibility was also not different (*p* = 0.97) when measured in the first protocol versus the second protocol (Fig. 4A). This was also seen when the different sexes and strains of mice were analyzed separately (Suppl. Fig. 1). Bland–Altman analyses also suggested no systematic bias between values of distensibility obtained at the first and second protocols (Fig. 4B). In fact, the bias was virtually zero, with the 95% lower and upper limits of agreement clearly overlapping zero (Fig. 4B).

**Fig. 5:**
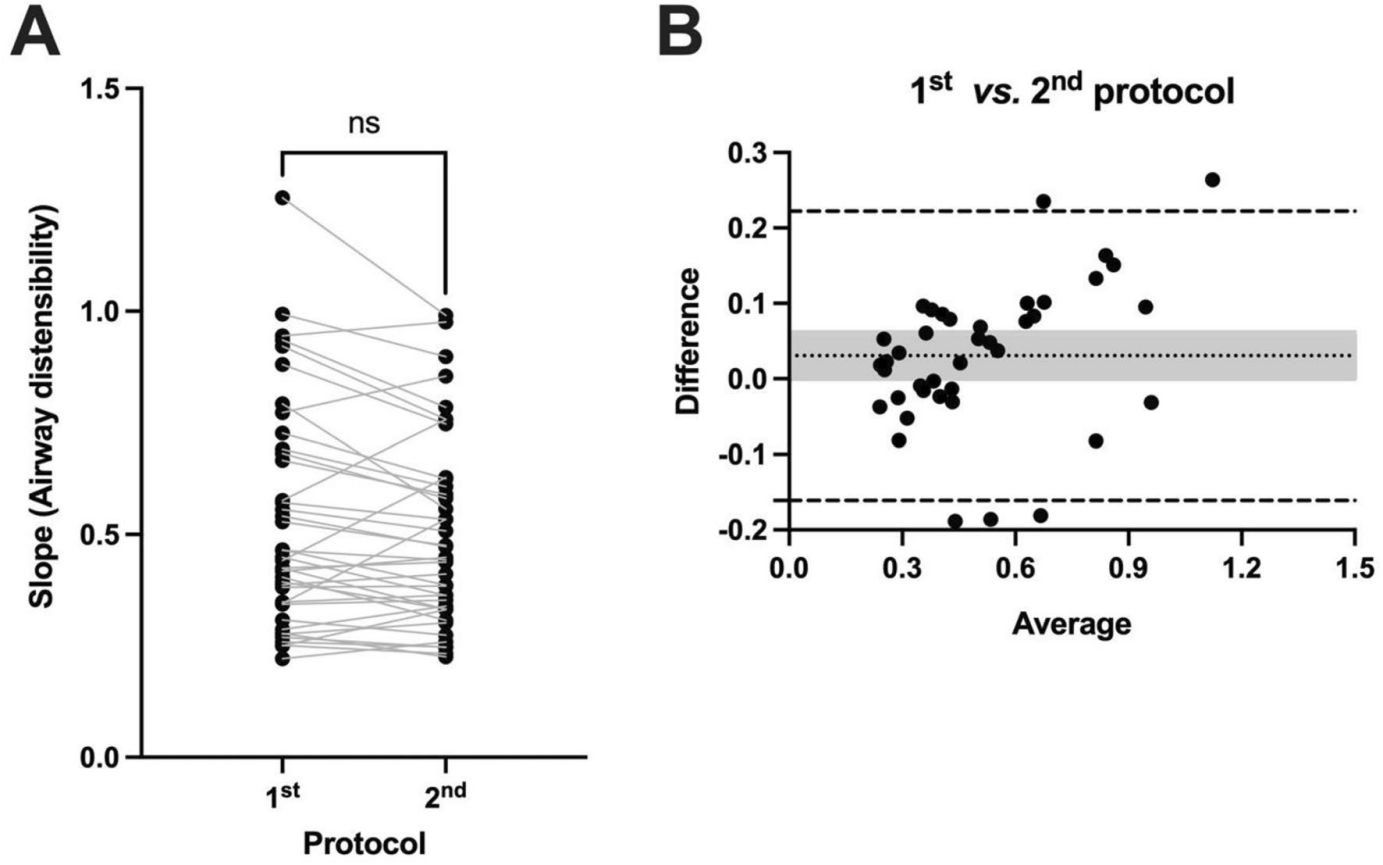
Reproducibility of airway distensibility when only data points in the ascending portion of the stepwise changes in pressure were used. **A** Airway distensibility was assessed twice in each mouse as described in the series of two protocols illustrated in Fig. 1. At each protocol, it was calculated as described in Fig. 3, except that only data points in the ascending portion of the stepwise changes in pressure (i.e., only the black symbols in Fig. 3) were used for tracing the linear regression and calculating its slope, thereby omitting all data points collected during the descending portion of the stepwise changes in pressure (i.e., omitting all the red symbols in Fig. 3). For each mouse, a line is connecting values obtained in the first and the second protocol. Based on a paired *t* test, airway distensibility between the first and the second protocols was not significantly different (ns). **B** Bland–Altman plot comparing the values of airway distensibility between the first and the second protocols when only data points in the ascending portion of the stepwise changes in pressure were used. Each symbol represents one mouse, relating the difference between the two protocols (airway distensibility measured during the 1st protocol minus airway distensibility measured during the 2nd protocol) on the *y*-axis with the average of both protocols (the sum of airway distensibility in the 1st and the 2nd protocol divided by two) on the *x*-axis. The dotted line is the bias, the dashed lines are the 95% upper and lower limits of agreement, and the gray-shaded area is covering the upper and lower 95% confidence intervals for the bias. *n* = 40 (10 female BALB/c, 10 male BALB/c, 10 female C57BL/6, and 10 male C57BL/6)

The analyses were repeated by using only the first four data points during the ascending portion of the protocol illustrated in Fig. 1 (thus, excluding again the point at the highest tested pressure but also all data points during the descending portion of the protocol). The ICC was then 0.91, still representing an excellent reproducibility for the values of distensibility between the first and second protocols. There was also no difference in the values of distensibility between the two protocols (Fig. 5A), although there was a numeric decrease in distensibility in the second versus the first protocol that was almost significant (*p* = 0.055). The lack of difference was also seen when the different sexes and strains of mice were analyzed separately (Suppl. Fig. 2). Bland–Altman analyses confirmed the lack of systematic bias, as the 95% lower and upper limits of agreement of the bias were still overlapping zero (Fig. 5B). Unless otherwise specified, the remaining analyses were conducted by calculating distensibility using both the ascending and descending points of the stepwise changes in pressure as described in Fig. 3.

**Fig. 6:**
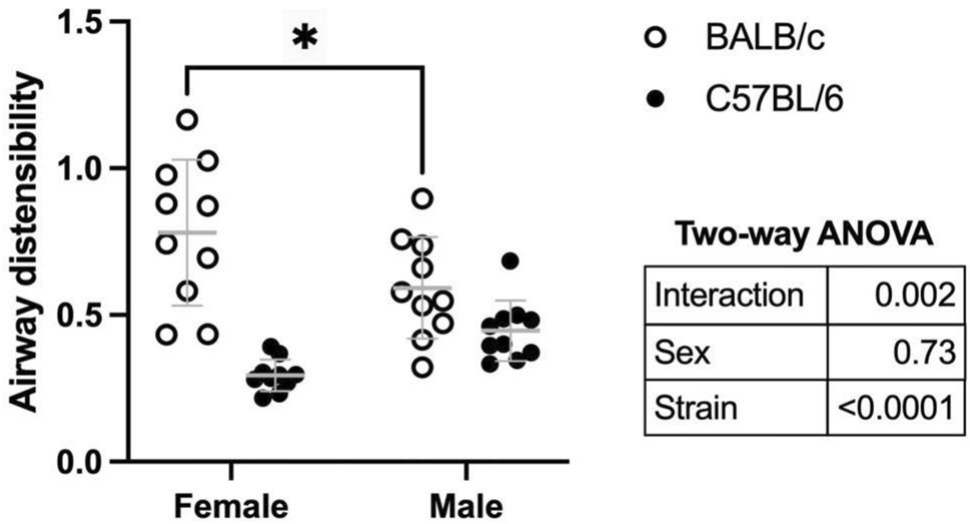
Sex and strain on airway distensibility. As per Fig. 4, airway distensibility was measured as described in Fig. 3. Results of the two-way ANOVA are shown in the table next to the graph. Since the interaction was significant, pairwise comparisons between sexes within each mouse strain were analyzed using a Sidak’s multiple comparisons test. Asterisks indicate statistically significant differences (* is *p* < 0.05). *n* = 10 female BALB/c, 10 male BALB/c, 10 female C57BL/6 and 10 male C57BL/6

Comparisons between mouse sexes and strains are depicted in Fig. 6. Airways of BALB/c mice were more distensible than airways of C57BL/6 mice (the effect of strain in the two-way ANOVA was *p* < 0.0001). Although sex had no effect overall (*p* = 0.73), there was a significant interaction between sex and mouse strain (*p* = 0.002). This was driven by the fact that airway distensibility in females was numerically greater than males in BALB/c mice, while, inversely, airway distensibility in males was numerically greater than females in C57BL/6. Post-hoc analyses suggested that the sex difference was only significant in BALB/c mice (*p* = 0.027), although a trend was also observed in C57BL/6 (*p* = 0.084). It is worth mentioning that the mouse strain difference and the sex–strain interaction were still observed when distensibility was calculated using data points from only the ascending portion of the first protocol illustrated in Fig. 1 (the strain effect and the interaction were then *p* < 0.0001 and *p* = 0.006, respectively) or from only the descending portion of the protocol (the strain effect and the interaction were then *p* < 0.0001 and *p* = 0.001, respectively), as well as by using data points from the second protocol or the average of both protocols using either the ascending portion only, the descending portion only, or both the ascending and descending portions of the stepwise changes in pressure (the strain effect in each of these 6 two-way ANOVAs was always *p* < 0.0001 and the interaction was always *p* < 0.007). However, post hoc analyses to evaluate the effect of sex within each mouse strain yielded different results depending on how distensibility was calculated, being sometimes significant only in BALB/c mice, sometimes significant in both strains, and sometimes only significant in C57BL/6 mice.

**Fig. 7:**
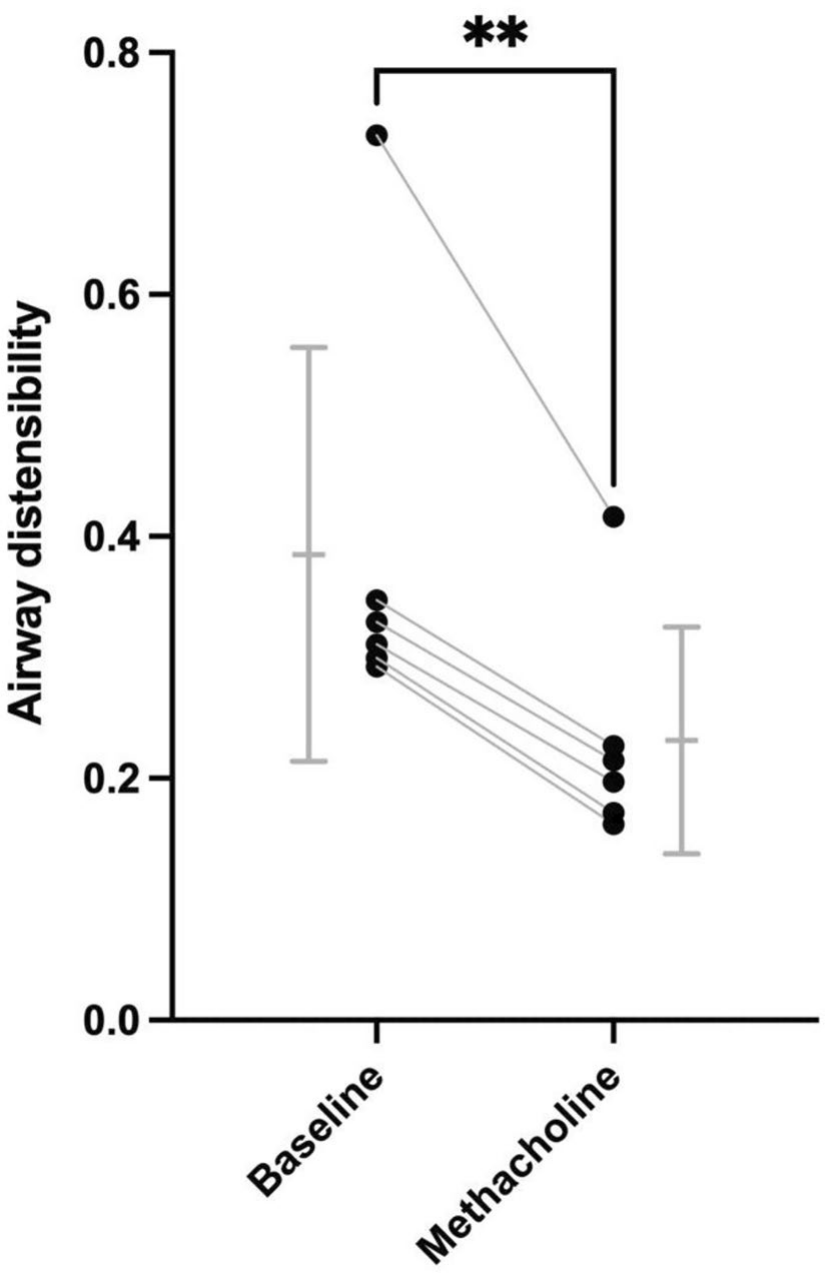
The effect of infused methacholine on airway distensibility in male C57BL/6 mice. Airway distensibility was assessed twice in each mouse, first at baseline and then during infused methacholine. As per Figs. 4 and 6, airway distensibility was measured as described in Fig. 3. For each mouse, a line is connecting values obtained at baseline and with infused methacholine. Asterisks are from a paired *t* test and indicate a statistically significant difference (** is *p* < 0.01). *n* = 6

The effect of infused methacholine on airway distensibility in male C57BL/6 mice is depicted in Fig. 7. At the lowest lung inflating pressure tested (i.e., 3 cmH_2_O), the infusion of methacholine first decreased *G*_*N*_ by 26% on average (3.89 ± 0.42 vs. 2.88 ± 0.31 mL/cmH_2_O·s, *p* = 0.001), confirming airway constriction. Methacholine then significantly decreased airway distensibility by 40% on average (0.39 ± 0.17 vs. 0.23 ± 0.09 mL/s, *p* = 0.005) (Fig. 7). It is worth mentioning that this methacholine effect was still observed when distensibility was calculated using only the first four points in the ascending portion (*p* = 0.028), or only the last four points in the descending portion (*p* = 0.003) of the stepwise changes in pressure illustrated in Fig. 1.

## Discussion

A method was developed to measure airway distensibility in mice. The method is quick (~ 6 min), reproducible, and sufficiently sensitive to detect a difference between BALB/c and C57BL/6 mice, as well as to detect an effect of sex that was mouse strain dependent. It was also markedly affected by the contraction of airway smooth muscle elicited by infused methacholine.

In humans, the oscillometric measurement of airway distensibility was shown to be reproducible [16], altered in respiratory diseases [11, 13, 15], and to change in response to treatments [13, 14]. In addition, the type of breathing maneuvers that are required to measure airway distensibility provides additional insightful indices. These include the critical closing volume, which is the lung volume at which reactance suddenly plummets during a deflationary maneuver; the latter being a sign of derecruitment (i.e., closure of small airways) that occurs at progressively greater lung volume with age and with severity of respiratory diseases [13, 26, 27].

An equivalent method to assess airway distensibility in mice was heretofore lacking. Owing to seminal developments in pre-clinical equipment over the last decades [28, 29], the precision with which oscillometry is used to measure respiratory mechanics in mice has long surpassed the one observed in humans [30]. Beyond quantifying resistance and reactance of the respiratory system at different frequencies, computational models can now be implemented on the impedance spectrum to provide parameters that can be interpreted with terms bearing an intuitive physiological meaning, such as airway resistance. Herein, we have used the constant-phase model [31] to deduce airway conductance (abbreviated *G*_*N*_) at different steps of lung inflating pressure. *G*_*N*_ is a parameter reflecting the conductance to airflow in the ensemble of airways in the lung and its changes with inflating pressure was shown to be a proper surrogate for the changes in airway volume [12].

The results clearly demonstrated that *G*_*N*_ increases with inflating steps in pressure and inversely decreases with deflating steps in pressure. Conveniently, these changes were approximately linear across a certain range of pressure. A single value of distensibility was then allocated per mouse, merely by calculating the slope of a linear regression that best fitted the *G*_*N*_ data within this pressure range. It is understood that airway distensibility is not the same across the entire range of lung pressure or volume. Traditionally, airway distensibility in humans was measured by fitting a polynomial line across the entire dataset and then calculating its derivative at any chosen pressure or volume [12, 13]. The latter is convenient as it can ascribe a value of airway distensibility at any given lung pressure or volume. One disadvantage though is that it suffers in reproducibility [16]. The other strategy used previously in humans was to trace a linear regression over different segments of the dataset [11, 14, 15], where it can be assumed that within each segment, the conductance data are changing approximately linearly with lung pressure or volume. This latter method was shown to be more reproducible [16]. It also provides an index of distensibility that spans a certain range of lung pressure or volume. This strategy is more alike the one used herein. It is, thus, understood that the mouse measure of airway distensibility presented in this study only pertains to a small range of low lung volumes, near functional residual capacity (FRC).

Importantly, the measurement was performed twice in the same mice with high reproducibility. This suggested that airway distensibility can be measured at least twice within the same mice. The method is, thus, suitable to compare airway distensibility before and after an intervention within the same mice. This also comes with a gain in statistical power due to paired analyses, which is also likely to reduce the number of mice and aligns with the 3Rs’ principles in research involving animals.

Alternative ways of calculating distensibility were also investigated. It was shown that similar data were obtained by only including data points in the ascending limb of the stepwise changes in pressure. This finding suggested that the duration of the method may be reduced by half (~ 3 min), as data would only need to be collected during the ascending limb of the stepwise changes in pressure. Yet it still came at the expense of a lesser reproducibility, as well as a risk of systematic bias and a statistical difference between the first and second assessments of airway distensibility, which were both borderline insignificant (Fig. 5). We think that the analyses are more robust when data points from both the ascending and the descending portions of the stepwise changes in pressure are used, not necessarily because the inflating and deflating motions are both required for accuracy, but probably because it doubles the number of data points and thereby reduces the random effect of the technical variability.

A quick and sensitive method was, thus, successfully developed to measure airway distensibility in mice. The method was then used to compare airway distensibility between mouse strains and sexes. BALB/c mice exhibited a greater airway distensibility than C57BL/6 mice. This is consistent with the stiffer lung of C57BL/6 versus BALB/c mice [17]. There was also a significant interaction between sex and mouse strain. While airway distensibility was greater in females than males in BALB/c mice, an inverse trend was observed in C57BL/6 mice. Interestingly, we have recently demonstrated in BALB/c mice that the thickness of the epithelium and the content of airway smooth muscle were both greater in males than females [18], perhaps explaining the lower airway distensibility in the former in this mouse strain. A similar study should be conducted to investigate whether these structural features also vary with sex in C57BL/6 mice and whether they are associated with a sexual dimorphism in airway distensibility.

Airway distensibility was also tested after activating the smooth muscle with infused methacholine. Although the contraction of smooth muscle is expected to stiffen the airway wall, its effect on airway distensibility was difficult to predict for at least two confounding phenomena. First, smooth muscle contraction causes airway narrowing. This forces the airway wall to operate on a more compliant part of the airway cross-sectional area–pressure curve and thereby to be strained further for any given swing in pressure. Indeed, this was previously shown in dogs in vivo using computed tomography [32]. In the latter, the changes in airway luminal area at varying lung transpulmonary pressure were measured in the presence or absence of infused methacholine. On the one hand, it was shown in relaxed airways that the maximal airway caliber is often achieved at low transpulmonary pressure (5–7 cmH_2_O) and, therefore, the changes in airway caliber from 5 to 10 cmH_2_O are sometimes very small. In constricted airways, on the other hand, the changes in airway caliber across the same range of pressure were often substantial because the caliber at a transpulmonary pressure of 5–7 cmH_2_O was nowhere near its maximum. Computational models also predicted a greater excursion of airway caliber during physiological swings in transpulmonary pressure when the smooth muscle is activated [33]. This is also an observation consistent with the greater swings in respiratory system resistance during tidal volume breathing in asthmatics versus non-asthmatics [34]; the former exhibiting a greater level of smooth muscle tone [13, 35].

Second, it is important to understand that, assuming Poiseuille flow, the changes in conductance of an airway are proportional to the square of the changes in luminal area (*G* = *A*^2^/8l*μπ*, where *G* is conductance, *l* is the length of the airway, *μ* is air viscosity, and *A* is area). Therefore, in the presence of smooth muscle-mediated airway constriction, smaller will be the initial airway caliber, smaller will be the change in conductance caused by any given airway dilation. In other words, based on this non-linear relationship between airway caliber and airflow conductance, interventions causing bronchoconstriction will tend to decrease distensibility, while the ones causing dilatation will tend to increase distensibility. In fact, when the data are reanalyzed in terms of normalized distensibility $\left[ {\left( {{{1} \mathord{\left/ {\vphantom {{1} {G_{N} }}} \right. \kern-0pt} {G_{N} }}} \right)*{{\Delta G_{N} } \mathord{\left/ {\vphantom {{\Delta G_{N} } {\Delta P}}} \right. \kern-0pt} {\Delta P}}} \right]$ instead of distensibility (Δ*G*_*N*_/Δ*P*), the effect of mouse strain is preserved but the effect of methacholine is lost (Suppl. Figs. 3, 4).

These confounding phenomena also have important implications. They imply that a change in distensibility does not always reflect a structural change in the mechanical properties of the airway wall, especially when it is triggered by a bronchoactive substance that either constricts or dilates airways. Nonetheless, airway distensibility was significantly decreased by methacholine in the present study. This result is consistent with Kelly and coworkers [13], showing that a bronchodilator drug actually exerted the opposite effect of methacholine in asthmatic individuals, effectively increasing airway distensibility. Kelly et al. [13] have also demonstrated that the effect of smooth muscle on airway distensibility is mainly perceived at low lung volumes (residual volume and FRC), which is about the range of lung volumes wherein airway distensibility was tested in the present study. This consistency between mice and humans regarding the contribution of smooth muscle reinforces the translational relevance of mouse studies on airway distensibility.

## Conclusion

Airway distensibility can be measured quickly in mice using oscillometry. It can also be measured twice in the same mouse with high reproducibility. The method was used herein to compare airway distensibility between sexes and two mouse strains. Airways of BALB/c mice were more distensible than C57BL/6 mice, and sex also affected distensibility in a mouse strain-dependent manner. Interpreting the role of smooth muscle contraction on airway distensibility is not as straightforward. Yet, in the range of low lung volumes wherein it was measured in the present study, airway constriction induced by the contraction of smooth muscle decreased airway distensibility. The method is now ready to be used for monitoring the progression of alterations in the mechanical properties of the airway wall in mouse models of respiratory diseases, as well as for testing the efficacy of novel treatments that aim at preventing or reversing these alterations by acting on airway wall remodeling, surface tension, or smooth muscle contraction.

### Supplementary Information

Below is the link to the electronic supplementary material.

Supplementary file1 (DOCX 457 KB)

## References

[1] Robichaud A, Fereydoonzad L, Limjunyawong N, Rabold R, Allard B, Benedetti A, Martin JG, Mitzner W (2017). Automated full-range pressure-volume curves in mice and rats. J. Appl. Physiol..

[2] Bossé Y (2019). The strain on airway smooth muscle during a deep inspiration to total lung capacity. J. Eng. Sci. Med. Diagn. Ther..

[3] Kelly VJ, Brown NJ, King GG, Thompson BR (2010). A method to determine in vivo, specific airway compliance, in humans. Med. Biol. Eng. Comput..

[4] Kelly VJ, Brown NJ, King GG, Thompson BR (2011). The bronchodilator response of in vivo specific airway compliance in adults with asthma. Ann. Biomed. Eng..

[5] Brown RH, Scichilone N, Mudge B, Diemer FB, Permutt S, Togias A (2001). High-resolution computed tomographic evaluation of airway distensibility and the effects of lung inflation on airway caliber in healthy subjects and individuals with asthma. Am. J. Respir. Crit. Care Med..

[6] Scichilone N, La Sala A, Bellia M, Fallano K, Togias A, Brown RH, Midiri M, Bellia V (2008). The airway response to deep inspirations decreases with COPD severity and is associated with airway distensibility assessed by computed tomography. J. Appl. Physiol..

[7] Pyrgos G, Scichilone N, Togias A, Brown RH (2011). Bronchodilation response to deep inspirations in asthma is dependent on airway distensibility and air trapping. J. Appl. Physiol..

[8] Diaz AA, Come CE, Ross JC, SanJoseEstepar R, Han MK, Loring SH, Silverman EK, Washko GR (2012). Association between airway caliber changes with lung inflation and emphysema assessed by volumetric CT scan in subjects with COPD. Chest.

[9] Bakker ME, Stolk J, Reiber JH, Stoel BC (2012). Influence of inspiration level on bronchial lumen measurements with computed tomography. Respir. Med..

[10] Benfante A, Bellia M, Scichilone N, Cannizzaro F, Midiri M, Brown R, Bellia V (2013). Airway distensibility by HRCT in asthmatics and COPD with comparable airway obstruction. COPD.

[11] Brown NJ, Salome CM, Berend N, Thorpe CW, King GG (2007). Airway distensibility in adults with asthma and healthy adults, measured by forced oscillation technique. Am. J. Respir. Crit. Care Med..

[12] Brown NJ, Thorpe CW, Thompson B, Berend N, Downie S, Verbanck S, Salome CM, King GG (2004). A comparison of two methods for measuring airway distensibility: Nitrogen washout and the forced oscillation technique. Physiol. Meas..

[13] Kelly VJ, Brown NJ, Sands SA, Borg BM, King GG, Thompson BR (2012). Effect of airway smooth muscle tone on airway distensibility measured by the forced oscillation technique in adults with asthma. J. Appl. Physiol..

[14] Kermode JA, Brown NJ, Hardaker KM, Farah CS, Berend N, King GG, Salome CM (2011). The effect of airway remodelling on airway hyper-responsiveness in asthma. Respir. Med..

[15] Baldi S, Dellaca R, Govoni L, Torchio R, Aliverti A, Pompilio P, Corda L, Tantucci C, Gulotta C, Brusasco V, Pellegrino R (2010). Airway distensibility and volume recruitment with lung inflation in COPD. J. Appl. Physiol..

[16] Mailhot-Larouche S, Lachance M, Bullone M, Henry C, Dandurand RJ, Boulet LP, Laviolette M, King GG, Farah CS, Bossé Y (2017). Assessment of airway distensibility by the forced oscillation technique: Reproducible and potentially simplifiable. Front. Physiol..

[17] Rojas-Ruiz A, Boucher M, Gill R, Gelinas L, Tom FQ, Fereydoonzad L, Graham P, Soliz J, Bosse Y (2023). Lung stiffness of C57BL/6 versus BALB/c mice. Sci. Rep..

[18] Gill R, Rojas-Ruiz A, Boucher M, Henry C, Bosse Y (2023). More airway smooth muscle in males versus females in a mouse model of asthma: A blessing in disguise?. Exp. Physiol..

[19] Bates JH, Irvin CG, Farré R, Hantos Z (2011). Oscillation mechanics of the respiratory system. Compr. Physiol..

[20] Hantos Z, Daroczy B, Suki B, Nagy S, Fredberg J (1992). Input impedance and peripheral inhomogeneity of dog lungs. J. Appl. Physiol..

[21] Sudy R, Fodor GH, Dos Santos RA, Schranc A, Tolnai J, Habre W, Petak F (2019). Different contributions from lungs and chest wall to respiratory mechanics in mice, rats, and rabbits. J. Appl. Physiol..

[22] Hirai T, McKeown KA, Gomes RF, Bates JH (1999). Effects of lung volume on lung and chest wall mechanics in rats. J. Appl. Physiol..

[23] Ito S, Lutchen KR, Suki B (2007). Effects of heterogeneities on the partitioning of airway and tissue properties in normal mice. J. Appl. Physiol..

[24] Lutchen KR, Hantos Z, Petak F, Adamicza A, Suki B (1996). Airway inhomogeneities contribute to apparent lung tissue mechanics during constriction. J. Appl. Physiol..

[25] Lexell JE, Downham DY (2005). How to assess the reliability of measurements in rehabilitation. Am. J. Phys. Med. Rehabil..

[26] Nilsen K, Gove K, Thien F, Wilkinson T, Thompson BR (2018). Comparison of two methods of determining lung de-recruitment, using the forced oscillation technique. Eur. J. Appl. Physiol..

[27] Kelly VJ, Sands SA, Harris RS, Venegas JG, Brown NJ, Stuart-Andrews CR, King GG, Thompson BR (2013). Respiratory system reactance is an independent determinant of asthma control. J. Appl. Physiol..

[28] Schuessler TF, Bates JH (1995). A computer-controlled research ventilator for small animals: design and evaluation. IEEE Trans. Biomed. Eng..

[29] McGovern TK, Robichaud A, Fereydoonzad L, Schuessler TF, Martin JG (2013). Evaluation of respiratory system mechanics in mice using the forced oscillation technique. J. Vis. Exp..

[30] Bates JH, Irvin CG (2003). Measuring lung function in mice: the phenotyping uncertainty principle. J. Appl. Physiol..

[31] Hantos Z, Daroczy B, Suki B, Nagy S, Fredberg JJ (1992). Input impedance and peripheral inhomogeneity of dog lungs. J. Appl. Physiol..

[32] Brown RH, Mitzner W (1996). Effect of lung inflation and airway muscle tone on airway diameter in vivo. J. Appl. Physiol..

[33] Hiorns JE, Jensen OE, Brook BS (2014). Nonlinear compliance modulates dynamic bronchoconstriction in a multiscale airway model. Biophys. J..

[34] Salome CM, Thorpe CW, Diba C, Brown NJ, Berend N, King GG (2003). Airway re-narrowing following deep inspiration in asthmatic and nonasthmatic subjects. Eur. Respir. J..

[35] Molfino NA, Slutsky AS, Julia-Serda G, Hoffstein V, Szalai JP, Chapman KR, Rebuck AS, Zamel N (1993). Assessment of airway tone in asthma. Comparison between double lung transplant patients and healthy subjects. Am. Rev. Respir. Dis..

