# Supplementary material for: A Quick Method to Assess Airway Distensibility in Mice
Source: Ann Biomed Eng. 2024 Apr 15;52(8):2193–202. doi: 10.1007/s10439-024-03518-9 (PMC11247055; doi:10.1007/s10439-024-03518-9)
Supplement: Supplementary file 1 — Supplementary file1 (DOCX 457 KB) [file 10439_2024_3518_MOESM1_ESM.docx]

**SUPPLEMENTAL**

**A quick method to assess airway distensibility in mice**

Rebecka Gill, Magali Boucher, Cyndi Henry, Ynuk Bossé

Institut Universitaire de Cardiologie et de Pneumologie de Québec (IUCPQ) – Université Laval, Québec (QC), Canada

**FIGURES & FIGURES LEGENDS**

**
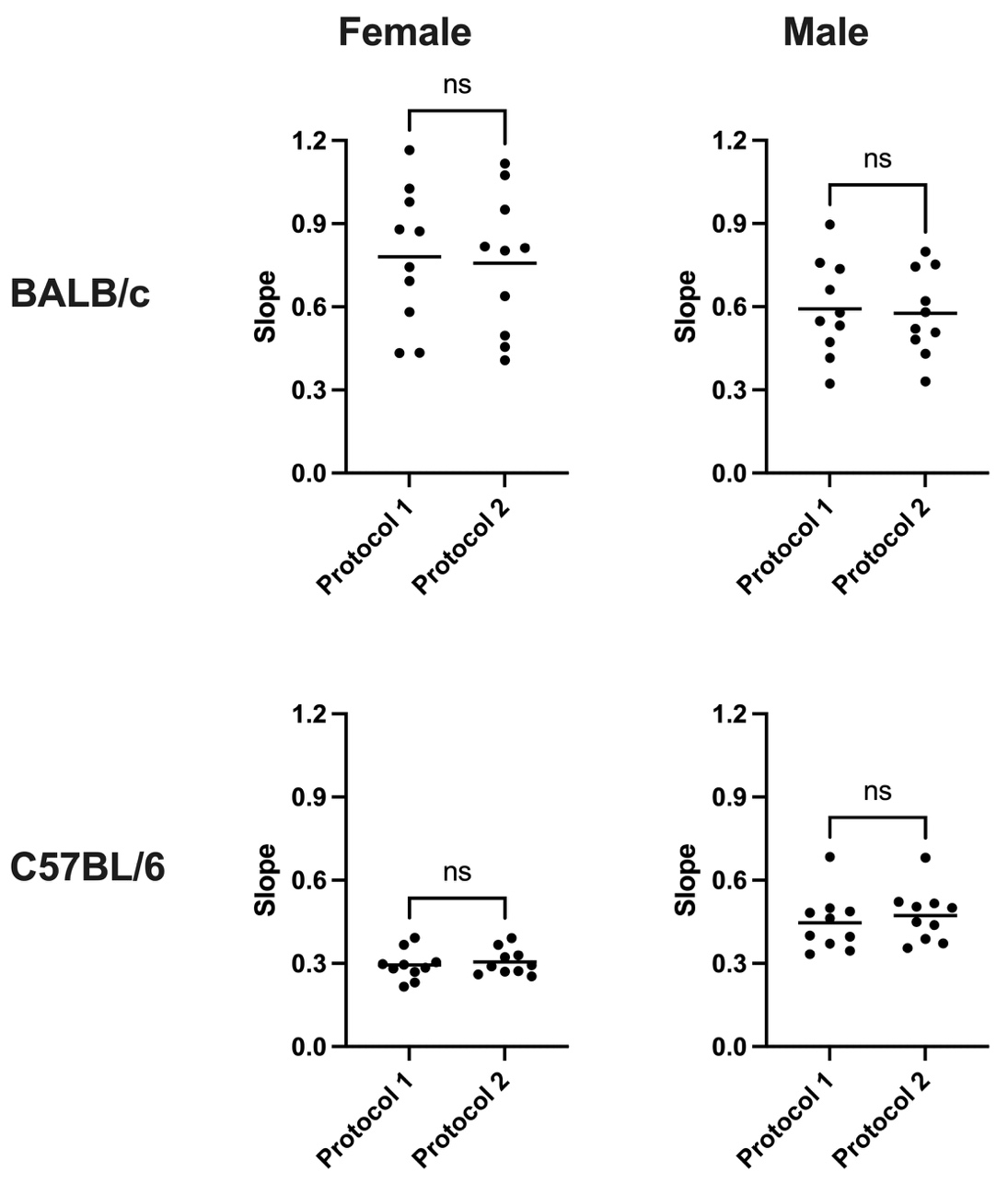
**

**Suppl. Figure 1.** Reproducibility of airway distensibility for either sex in both mouse strains. Airway distensibility, measured as described in Figure 3, was assessed twice in each mouse as described in the series of two protocols illustrated in Figure 1. Based on paired t-tests, airway distensibility between the first and the second protocols was not significantly different for mice of either sex in both mouse strains. ns stands for non-significant. n = 10 female BALB/c, 10 male BALB/c, 10 female C57BL/6 & 10 male C57BL/6


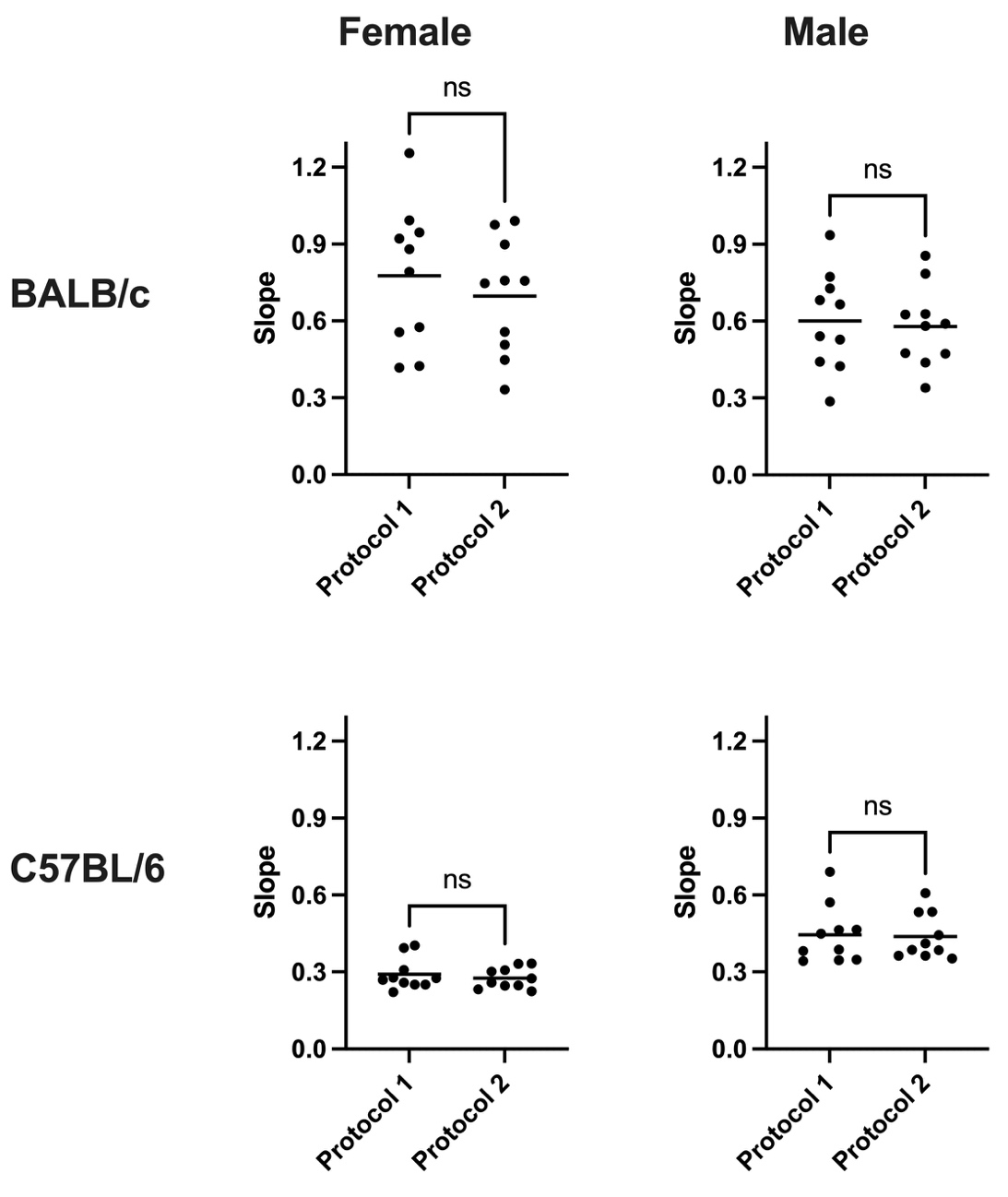


**Suppl. Figure 2.** Reproducibility of airway distensibility for either sex in both mouse strains when only data points in the ascending portion of the stepwise changes in pressure were used. Airway distensibility was assessed twice in each mouse as described in the series of two protocols illustrated in Figure 1. At each protocol, it was calculated as described in Figure 3, except that only data points in the ascending portion of the stepwise changes in pressure (*i.e.*, only the black symbols in Figure 3) were used for tracing the linear regression and calculating its slope; thereby omitting all data points collected during the descending portion of the stepwise changes in pressure (*i.e.*, omitting all the red symbols in Figure 3). Based on paired t-tests, airway distensibility between the first and the second protocols was not significantly different for mice of either sex in both mouse strains. ns stands for non-significant. n = 10 female BALB/c, 10 male BALB/c, 10 female C57BL/6 & 10 male C57BL/6


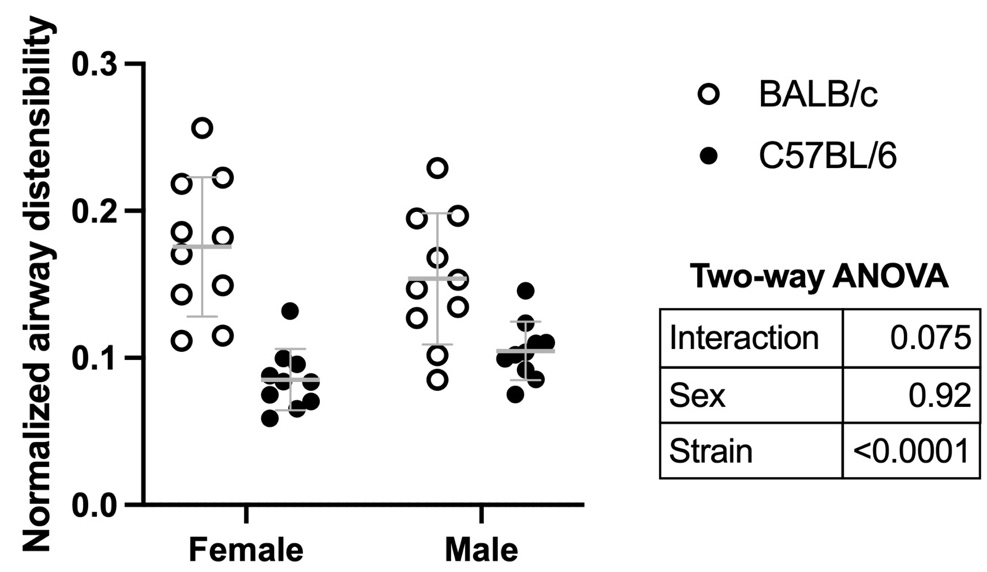


**Suppl. Figure 3.** Sex and strain on normalized airway distensibility. These are the same data as Figure 6 in the main document, but reanalyzed in terms of normalized distensibility [(1/G_N_)*ΔG_N_/ΔP] instead of distensibility (ΔG_N_/ΔP). Results of the two-way ANOVA are shown in the table next to the graph. n = 10 female BALB/c, 10 male BALB/c, 10 female C57BL/6 & 10 male C57BL/6


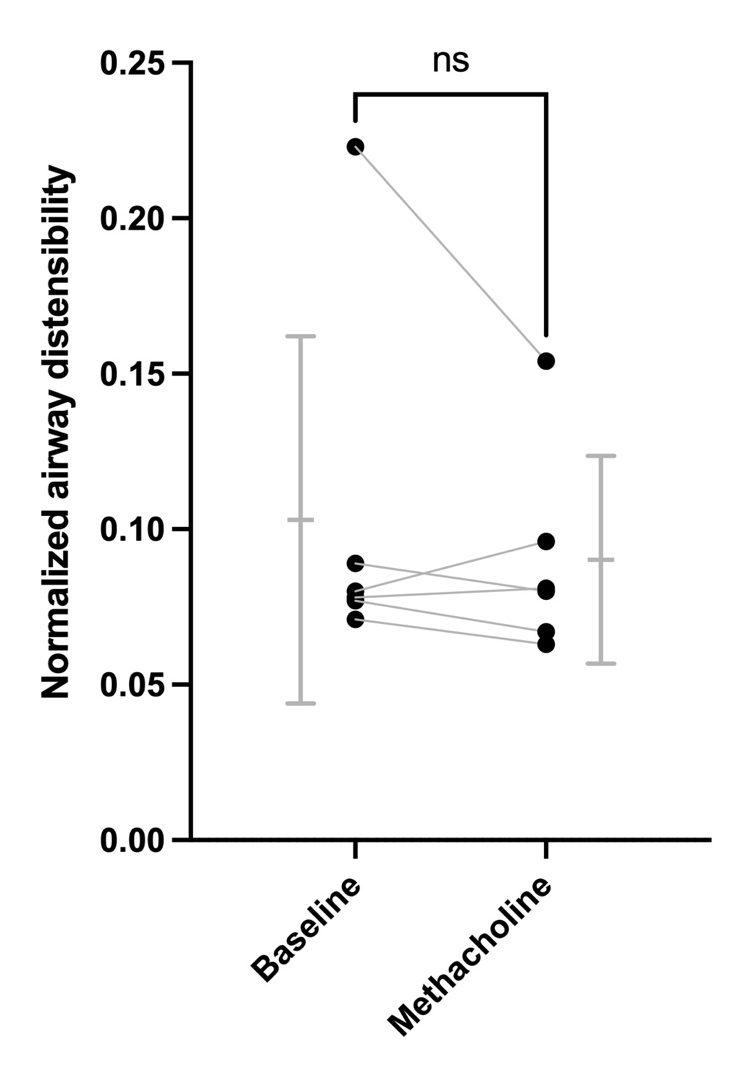


**Suppl. Figure 4.** The effect of infused methacholine on normalized airway distensibility in male C57BL/6 mice. These are the same data as Figure 7 in the main document, but reanalyzed in terms of normalized distensibility [(1/G_N_)*ΔG_N_/ΔP] instead of distensibility (ΔG_N_/ΔP). Airway distensibility was assessed twice in each mouse, first at baseline and then during infused methacholine. For each mouse, a line is connecting values obtained at baseline and with infused methacholine. Based on a paired t-test, normalized airway distensibility without and with infused methacholine was not significantly different (ns). n = 6
